# Supplementary material for: Autocrine TGF-β-positive feedback in profibrotic AT2-lineage cells plays a crucial role in non-inflammatory lung fibrogenesis
Source: Nat Commun. 2023 Aug 31;14:4956. doi: 10.1038/s41467-023-40617-y (PMC10471635; doi:10.1038/s41467-023-40617-y)
Supplement: Supplementary file 1 — Supplementary Information [file 41467_2023_40617_MOESM1_ESM.pdf]

Supplementary Information

Title: Autocrine TGF-β-positive feedback in profibrotic AT2-lineage cells plays a crucial role in non-inflammatory lung fibrogenesis

Supplementary methods

Induction of lung fibrosis by intratracheal administration of bleomycin

To induce lung parenchymal fibrosis, mice were anesthetized by isoflurane (Pfizer, NY, USA) and administered a single intratracheal injection of 1.2 or 2.0 mg per kg bleomycin sulphate (Nippon Kayaku, Tokyo, Japan) in sterile PBS using a MicroSprayer™ (Penn-Century, PA, USA).

Assessment of lung mCT

Mice were anesthetized by isoflurane and scanned by a mCT device (CosmoScan GX II, Rigaku). Lung imaging was performed with respiratory synchronization as appropriate. Field of view was set at 20 mm and a total of 512 slices per a mouse were evaluated. Grading of lung injury severity on whole lung mCT images was defined as followings. Grade 0: Normal lung density; Grade 1: <10% area of ground-glass attenuation (GGA)/consolidation; Grade 2: >10% area of GGA and <10% area of consolidation (typically with traction bronchiectasis); Grade 3: 10-30% area of consolidation; Grade 4: 30-50% area of consolidation; Grade 5: 50-70% area of consolidation; Grade 6: >70% area of consolidation.

Bronchoalveolar lavage (BAL) of mouse lungs

Mice were sacrificed by carbon dioxide with blood removal and perfused with 5 mL of saline solution thorough the right ventricle. After puncture of the middle trachea with a 22 G needle, the catheter was

inserted into the trachea. Then, 0.8 mL of sterile PBS was loaded and collected for three times. A total of 2 mL of BAL fluid was used. Cell number was manually counted using a hemacytometer.

Preparation of mouse primary cells and FACS sorting

Mice were sacrificed by carbon dioxide and perfused with 5 mL of saline solution thorough the right ventricle. 0.8 mL of protease solution [Collagenase type I (450 U·mL^{-1}, Worthington), Elastase (1 U·mL^{-1}, Worthington), Dispase (5 U·mL^{-1}, Corning), DNaseI (0.05 mg·mL^{-1}, SIGMA), Trypsin (0.025%, Thermo Fisher Scientific) in DMEM/F12] was intratracheally injected and the trachea was tied with a string. After incubation for 5 min at 37 °C on a dish, only lung lobes were extracted. These lobes were chopped to small pieces with a sterile blade and incubated in a total of 4 mL of the protease solution for 30 min at 37 °C with a rotator. Then the tissues/cells were dissociated by repetitive pipetting and 4 mL of PBS containing 10 % FBS was added. And then cells were filtered through 100 μm and 40 μm strainer. After a centrifugation (400*g*, 5 min), the cells were incubated with RBC lysis buffer (BioLegend) at room temperature for 3 min. Next, the cells were resuspended in PBS containing 3 % FBS and centrifuged (400g, 5 min). After that, the cells were stained with antibodies (Supplementary Data) and sorted by FACS AriaII (BD Biosciences). FACSDiva v8.0.2 (BD Bioscineces) was used to collect data and FlowJo v10.8.1 was used to analyze all FACS data.

Preparation of human primary AT2 cells and MACS sorting

Human lung tissues were obtained from three patients who received surgery for suspected early-stage lung cancer and did not have apparent emphysematous nor fibrotic changes on their CT images. Only normal lung specimens away from lung cancer lesion were used for experiments. These specimens were washed using sterile PBS for three times and then chopped to small pieces with a sterile blade after trimming out the pleura and incubated in 4 mL of the protease solution for 60 min at 37 °C with a rotator. Then the tissues or cells were dissociated by repetitive pipetting and a 4 mL of PBS containing 10 % FBS was added. And then cells were filtered through 100 μm and 40 μm strainer. After a centrifugation (400g, 5 min), the cells were incubated with RBC lysis buffer (BioLegend) at room temperature for 3 min. Next, the cells were resuspended in PBS containing 3 % FBS and centrifuged (400g, 5 min). After that, the cells were stained with anti-HTII-280 antibody and subsequently with anti-mouse IgM MicroBeads (Miltenyi Biotec), and then, sorted by MACS according to manufacturer's instructions.

Total RNA isolation, cDNA preparation, and quantitative RT-PCR

Total RNA was purified with ISOGEN (NIPPON GENE) according to manufacturer's instructions. Total RNA was reverse transcribed to cDNA by SuperScript III First-strand synthesis system (INVITROGEN) according to manufacturer's instructions. Quantitative RT-PCR was performed using primer sets shown in Supplementary Data with Thunderbird SYBR qPCR mix (TOYOBO) on QuantStudio5 Real-time PCR machine (Thermo Fisher Scientific). PCR cycling parameters were

95 °C for 10 min (one cycle); 95 °C for 15 sec and 60 °C for 60 sec (40 cycles). *Gapdh*/*GAPDH* (glyceraldehyde 3-phosphate dehydrogenase) was used for normalization of gene expression as a house-keeping gene. Fold-changes in expression of targeted genes were calculated using $2^{-\Delta\Delta Ct}$ method. Heatmap visualization was performed using the GraphPad Prism 9.

.

Bulk RNA-seq of mouse alveolar organoids

Total RNAs were isolated from mouse alveolar organoids derived from the lung tissues of four independent mice using ISOGEN (NIPPON GENE) with on-column DNase digestion, according to the manufacturer's instructions. Quality control of the total RNAs were performed with Bioanalyzer 2100 (Agilent Technologies). Libraries were prepared with Illumina Stranded mRNA Prep, Ligation (Illumina) kit, and the sequencing was performed on Illumina HiSeq X at GENEWIZ Inc. to acquire paired-end 150 nt reads. Illumina adaptor sequences and low-quality bases were removed from the sequencing reads with Trim galore ver. 0.6.5 (https://www.bioinformatics.babraham.ac.uk/projects/trim_galore/). The trimmed RNA-seq reads were aligned to the UCSC GRCm39/mm39 genome sequences using HISAT2 ver. 2.2.1 with default parameters. Gene expression quantification was performed using StringTie ver. 2.1.4, and normalization was performed using edgeR ver. 3.32.1. Likelihood ratio tests for differential expression were performed with the edgeR glmFit and glmLRT functions, and FDR was calculated using the

Benjamini-Hochberg method. Heatmap visualization was performed using the web-based tool iDEP ver. 0.95 (http://bioinformatics.sdstate.edu/idep/). Pathway analysis was performed at Enrichr (https://maayanlab.cloud/Enrichr/; MSigDB Hallmark 2020[1]) using the list of differentially expressed genes identified in BLM-treated alveolar organoids with FDR <0.05 and fold change >1.2 compared to the control (Supplementary Data).

Preparation of lung or organoid sections

Lungs and alveolar organoids were fixed with 4% paraformaldehyde (PFA) at 4 °C for overnight for lungs and for 1 hr for organoids, respectively, embedded in paraffin or OCT (for frozen sections) and sectioned at 6-8 μm. For lung sections, at least three lobes with maximum area were analyzed in each mouse. For organoid sections, at least five organoids per one mouse/human in each situation were imaged. Images were taken by Zeiss 710 confocal microscope (Carl Zeiss).

Collagen staining of lung sections and the quantification

The area of lung fibrosis, defined as collagen deposition positively stained with Sirius Red in at least three lobes in each mouse, was quantified using ImageJ software and the mean value was used as data from one mouse. All data were integrated from those of six independent mice. Images were taken by IX83 microscope (OLYMPUS).

Immunohistochemistry

Sections were stained with primary antibodies listed in Supplementary Data. Secondary antibodies directly conjugated with Alexa Fluor 488/594/647 were used for multi-color imaging. DAPI (Nacalai) was used for nuclear counter staining. Images were obtained with Zeiss 710 confocal microscope (Carl Zeiss). Staining-positive area and staining positivity in the target cells were analyzed in at least three lobes in each mouse using ImageJ software and the mean value was used as data from one mouse. All data were integrated from those of three independent mice.

Staining of senescence-associated β-galactosidase

Alveolar organoids were fixed with 2% PFA for 10 min at room temperature. After removal of the PFA, the organoids were stained with senescence-associated β-galactosidase for overnight using Senescence Detection Kit (BioVision). Images were taken by IX83 microscope (OLYMPUS).

Proximity ligation in situ hybridization (PLISH)

All procedures for PLISH are based on a recent study[2] using mouse frozen lung sections. Probes for each gene were designed as appropriate (Supplementary Data). Images were obtained with Zeiss 710 confocal microscope (Carl Zeiss). Staining positivity in the target cells were analyzed in at least three lobes in each mouse using ImageJ software and the mean value was used as data from one mouse. All data were integrated from those of three independent mice.

Hydroxyproline assay

Total left lung was collected in each mouse and minced using BEADS CRUSHER μT-12 (TAITEC). Quantification of hydroxyproline was performed by Total Collagen Assay Kit (QuickZyme Biosciences B.V.) according to manufacturer's instructions.

Western blotting

Cells were immersed in 0.1 mL RIPA buffer (Nacalai) with Protease Inhibitor Cocktail (Nacalai) and Phosphatase inhibitor Cocktail (Nacalai) and then were sonicated for 5 min. After protein quantification of each sample, SDS-PAGE Sample Buffer (Tokyo Kasei) was added and stored at -80°C. After 2 min of boiling at 95°C, 10 or 15 μg protein per lane was run on Mini-protean TGX gel 4-15% (Bio-Rad). Then the proteins were transferred to PVDF membrane (Bio-Rad). The membranes were cut for the target region and blocked in ECL Prime Blocking Agent (GE Healthcare) for 30 min at room temperature. Next, the membranes were probed with primary antibodies (Supplementary Data) for 1 h at room temperature. After washing with PBS for 3 times, the membranes were immunoprobed with secondary antibodies (Supplementary Data) for 1 h at room temperature. Finally, membranes were washed with PBS for 3 times, and the antigen-antibody complexes were visualized with the Chemi-Lumi One L or Ultra (Nacalai) and imaged by Las-3000 mini (FUJIFILM). Band intensity was

normalized using GAPDH or using the total form when analyzing phosphorylated target proteins. The raw images of membranes/blots are included in Source Data.

Chromatin immunoprecipitation-sequencing (ChIP-seq) analysis

Alveolar organoids treated with or without BLM for 24 h were used for the analysis. Organoid cells in Matrigel were dissociated by TrypLE Express Enzyme (ThermoFisher Scientific) for 30 min at 37°C, followed by centrifugation. Cells were fixed in a PBS (-) solution containing 1 % formaldehyde and 2 mM DSG (disuccinimidyl glutarate; ThermoFisher Scientific) for 10 min in a water bath at 25°C. The crosslinking reaction was quenched by adding glycine to the final concentration of 125 mM. Chromatin immunoprecipitation (ChIP) was performed as previously described[3] except that, RIPA buffer was used instead of LB3 lysis buffer at permeabilization and sonication steps. Chromatin lysate from 1-5 × 10^7 organoid cells was prepared using the Covaris S220 sonicator (Covaris) in 1 mL of RIPA buffer supplemented with protease inhibitor cocktail (ThermoFisher Scientific) in a milliTUBE (Covaris) under the following condition; PIP: 175, duty factor: 15%, cycles per burst: 200, at 7°C bath temperature, and 10 min of duration. Immunoprecipitation was performed using 1 mL of the chromatin lysate supplemented with protease inhibitor cocktail which corresponds to 1-1.5 × 10^7 cells, and 20 µL of Protein A beads (ThermoFisher Scientific) bound with 15 µL of the anti-p53 rabbit monoclonal antibody (Cell Signaling; #32532, Lot: 2, 177 $\mu g \cdot mL^{-1}$). Illumina compatible libraries were prepared from 20 ng of input DNA or the 2-4 ng ChIP DNA after size selection for 50-400 bp DNA fragments

by AMPure XP beads, using the KAPA LTP Library Preparation kit (KAPA Biosystems) and TruSeq DNA UD Indexes (Illumina). ChIP libraries were sequenced on Illumina NextSeq 2000, using the P2 100-cycle flowcell (Illumina), and single-end 101 base sequencing reads were obtained. Adapter sequences and low-quality bases were removed from the sequencing reads with Trim galore ver. 0.6.7 with '-e 0.1 --nextseq 30' parameters (https://www.bioinformatics.babraham.ac.uk/projects/trim_galore/). The trimmed reads were aligned to the UCSC mm39 genome sequences using Bowtie2 ver. 2.5.1[4] with default parameters. Filtering of uniquely mapped reads was performed using -F '[XS] == null and not unmapped and not duplicate' in Sambamba ver. 0.8.2[5]. Peaks were called using MACS3 ver. 3.0.0b1[6] with the significance cut-off q-value = 0.01 for each sample (peak number: CTRLrep1 = 50430; CTRLrep2 = 48743; BLMrep1 = 39577; BLMrep2 = 41451). Identification of peaks that were significantly enriched in the BLM-treated samples was calculated using the DiffBind ver. 3.8.4[7] with edgeR ver. 3.40.2[8] and filtered with FDR <0.05. To associate peaks with genes, peak positions were converted to mm10 and then analyzed on the GREAT ver. 4.0.4 website[9], and pathway enrichment analysis was performed using Enrichr (https://maayanlab.cloud/Enrichr/; MSigDB Hallmark 2020[1]).

Re-analysis of datasets of single-cell RNA-seq

Re-analyses for single-cell RNA-seq datasets were performed using R studio software using the Seurat package (https://satijalab.org/seurat/index.html). TGF-β signaling in each cell cluster was analyzed using CellChat[10].

Supplementary references

1 Liberzon, A. *et al.* The Molecular Signatures Database (MSigDB) hallmark gene set collection. *Cell Syst* **1**, 417-425, doi:10.1016/j.cels.2015.12.004 (2015).

2 Nagendran, M., Riordan, D. P., Harbury, P. B. & Desai, T. J. Automated cell-type classification in intact tissues by single-cell molecular profiling. *Elife* **7**, doi:10.7554/eLife.30510 (2018).

3 Kadota, M. *et al.* CTCF binding landscape in jawless fish with reference to Hox cluster evolution. *Sci Rep* **7**, 4957, doi:10.1038/s41598-017-04506-x (2017).

4 Langmead, B. & Salzberg, S. L. Fast gapped-read alignment with Bowtie 2. *Nat Methods* **9**, 357-359, doi:10.1038/nmeth.1923 (2012).

5 Tarasov, A., Vilella, A. J., Cuppen, E., Nijman, I. J. & Prins, P. Sambamba: fast processing of NGS alignment formats. *Bioinformatics* **31**, 2032-2034, doi:10.1093/bioinformatics/btv098 (2015).

6 Zhang, Y. *et al.* Model-based analysis of ChIP-Seq (MACS). *Genome Biol* **9**, R137, doi:10.1186/gb-2008-9-9-r137 (2008).

7 Ross-Innes, C. S. *et al.* Differential oestrogen receptor binding is associated with clinical outcome in breast cancer. *Nature* **481**, 389-393, doi:10.1038/nature10730 (2012).

8 Robinson, M. D., McCarthy, D. J. & Smyth, G. K. edgeR: a Bioconductor package for differential expression analysis of digital gene expression data. *Bioinformatics* **26**, 139-140, doi:10.1093/bioinformatics/btp616 (2010).

9 McLean, C. Y. *et al.* GREAT improves functional interpretation of cis-regulatory regions. *Nat Biotechnol* **28**, 495-501, doi:10.1038/nbt.1630 (2010).

10 Jin, S. *et al.* Inference and analysis of cell-cell communication using CellChat. *Nat Commun* **12**, 1088, doi:10.1038/s41467-021-21246-9 (2021).

Supplementary Fig. 1. Supporting data of Fig. 1.

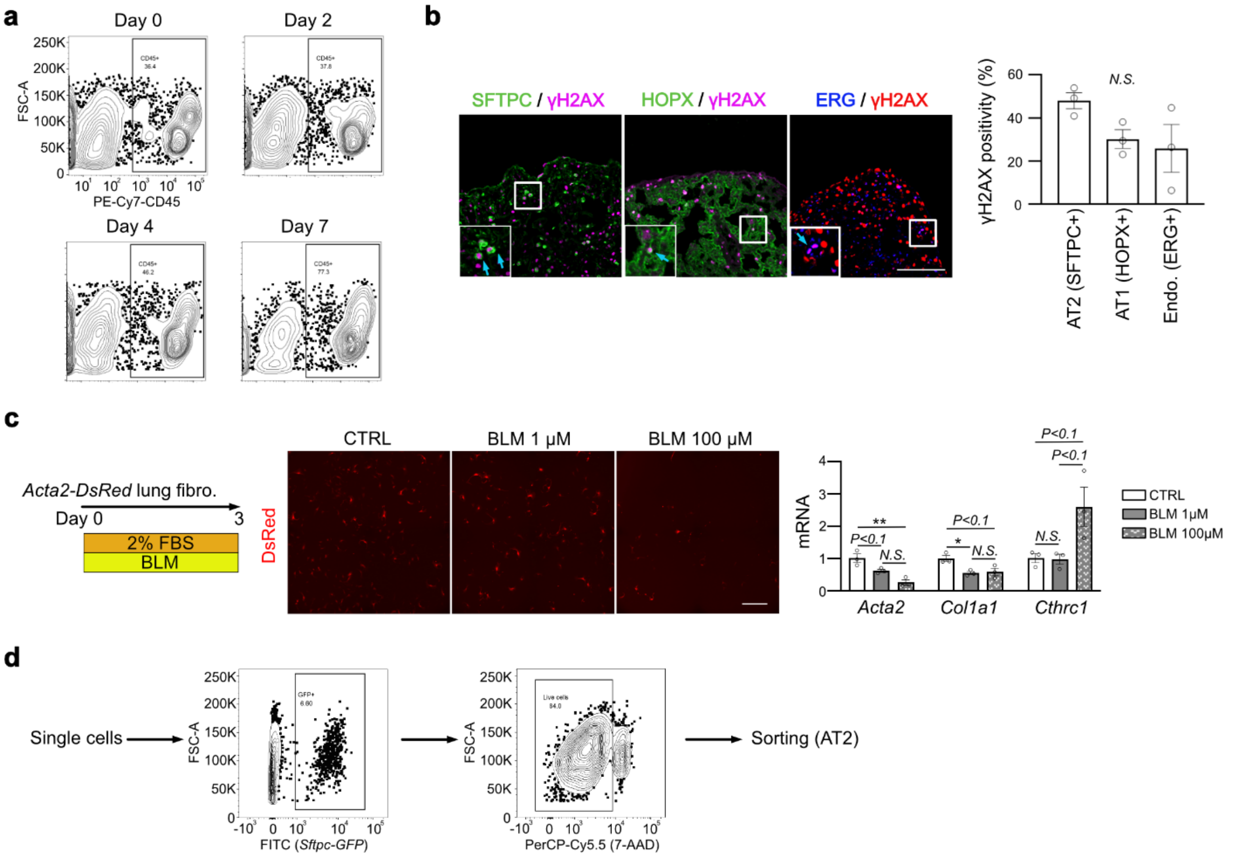

a) FACS gating strategy for primary hematopoietic cells from mouse lungs.

b) Representative images of lobe sections treated with BLM on days 2 stained with anti-SFTPC (for AT2)/HOPX (for AT1)/ERG (for endothelial cell) and anti-γH2AX antibodies (*scale bar: 100 μm*). Blue arrows indicate γH2AX-posi. AT2/AT1/endothelial cells at day 2. The right graph shows the quantification of γH2AX-positivity in each cell type at subpleural area (within a distance of 300 μm from the pleura). Data represent the mean ± SEM of results obtained from three independent mice and are compared using one-way analysis of variance with Bonferroni correction.

c) Evaluation of BLM-induced fibroblast-to-myofibroblast differentiation using *in vitro* single culture of FACS-sorted primary lung fibroblasts from *Acta2-DsRed* mice. Representative images of (myo)fibroblasts with raw DsRed fluorescence (*scale bar: 200 μm*) and the comparison of mRNA expression levels evaluated by qRT-PCR are shown. Data represent the mean ± SEM of results obtained from three independent mice. $*P<0.05$, $**P<0.01$, $***P<0.001$ (one-way analysis of variance with Bonferroni correction).

d) FACS gating strategy for primary AT2 cells from $Sftpc^{CreERT2}$:$Rosa26^{mTmG}$ mouse lungs.

Supplementary Fig. 2.: Supporting data of Fig. 2.

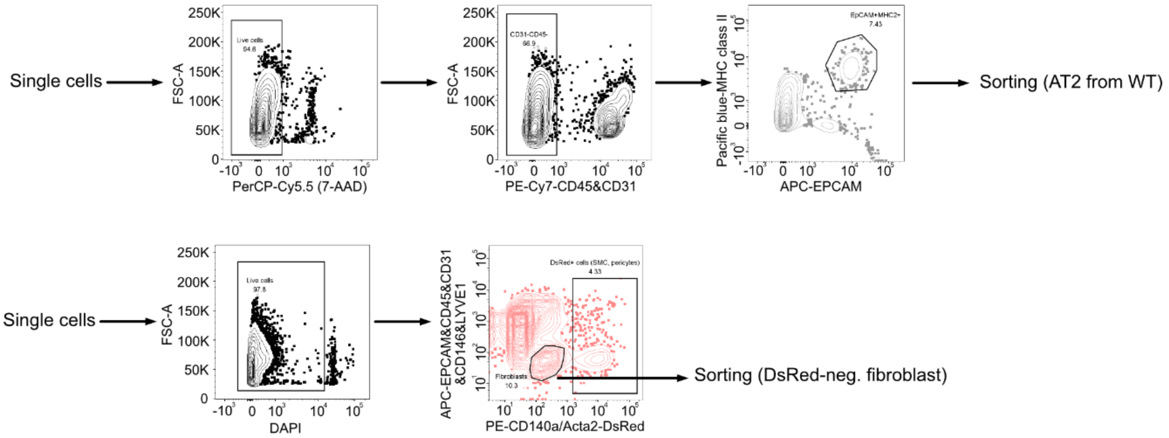

FACS gating strategy for primary AT2 cells from wild-type mouse lungs (*upper*) and primary fibroblasts from *Acta2-DsRed* mouse lungs (*lower*).

Supplementary Fig. 3. Supporting data of Fig. 3.

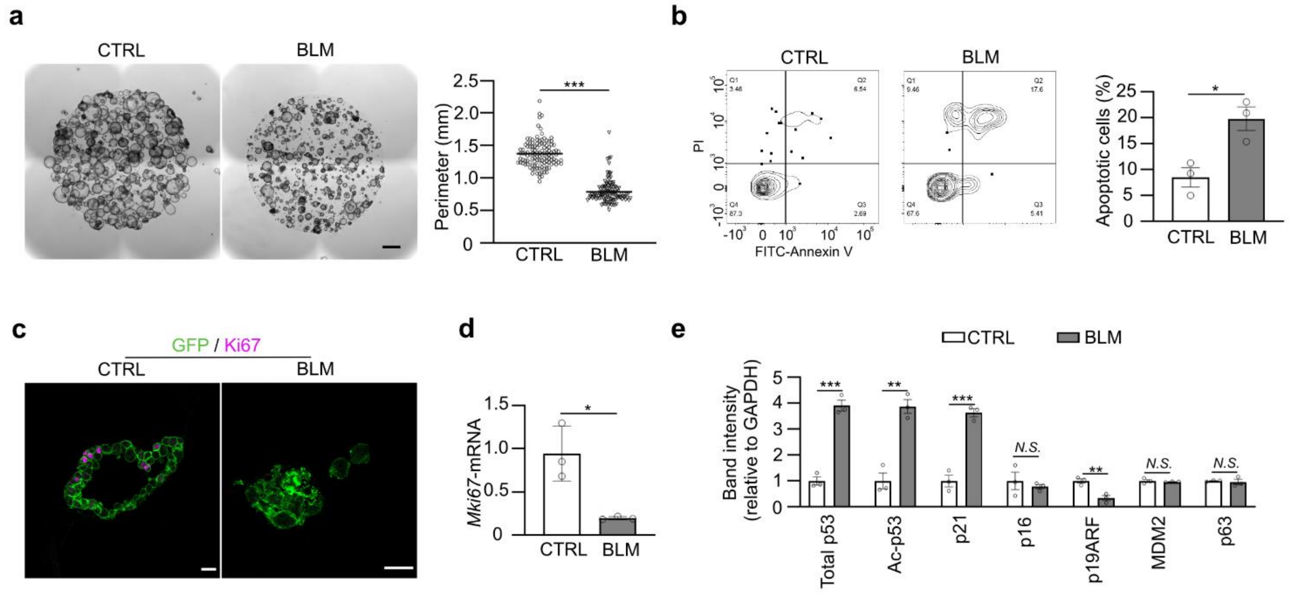

a) Representative images of alveolar organoids in bright field (*scale bar: 500 µm*) on culture day 8 treated with BLM (100 µM, 24 h) and the size quantification. Data represent the mean with individual values obtained from three independent mice. ****P* <0.001 (unpaired, two-tailed Student's t-test).
b) Quantification of Annexin V-positive apoptotic cells in alveolar organoids on culture day 8 treated with BLM (100 µM, 24 h) using FACS. Data represent the mean ± SEM of results obtained from three independent mice. **P* <0.05 (unpaired, two-tailed Student's t-test).
c) Representative images of organoid sections stained with anti-GFP and anti-Ki67 antibodies (*scale bar: 20 µm*).
d) Comparison of Mki67 mRNA expression level evaluated by qRT-PCR using alveolar organoids on culture day 8 treated with BLM (100 µM, 24 h). Data represent the mean ± SEM of results obtained from three independent mice. **P* <0.05 (unpaired, two-tailed Student's t-test).
e) Quantification of the protein expression of alveolar organoids via western blotting. Data represent the mean ± SEM of results obtained from three independent mice. ***P* <0.01, ****P* <0.001 (unpaired, two-tailed Student's t-test).

Supplementary Fig. 4. Supporting data of Fig. 4.

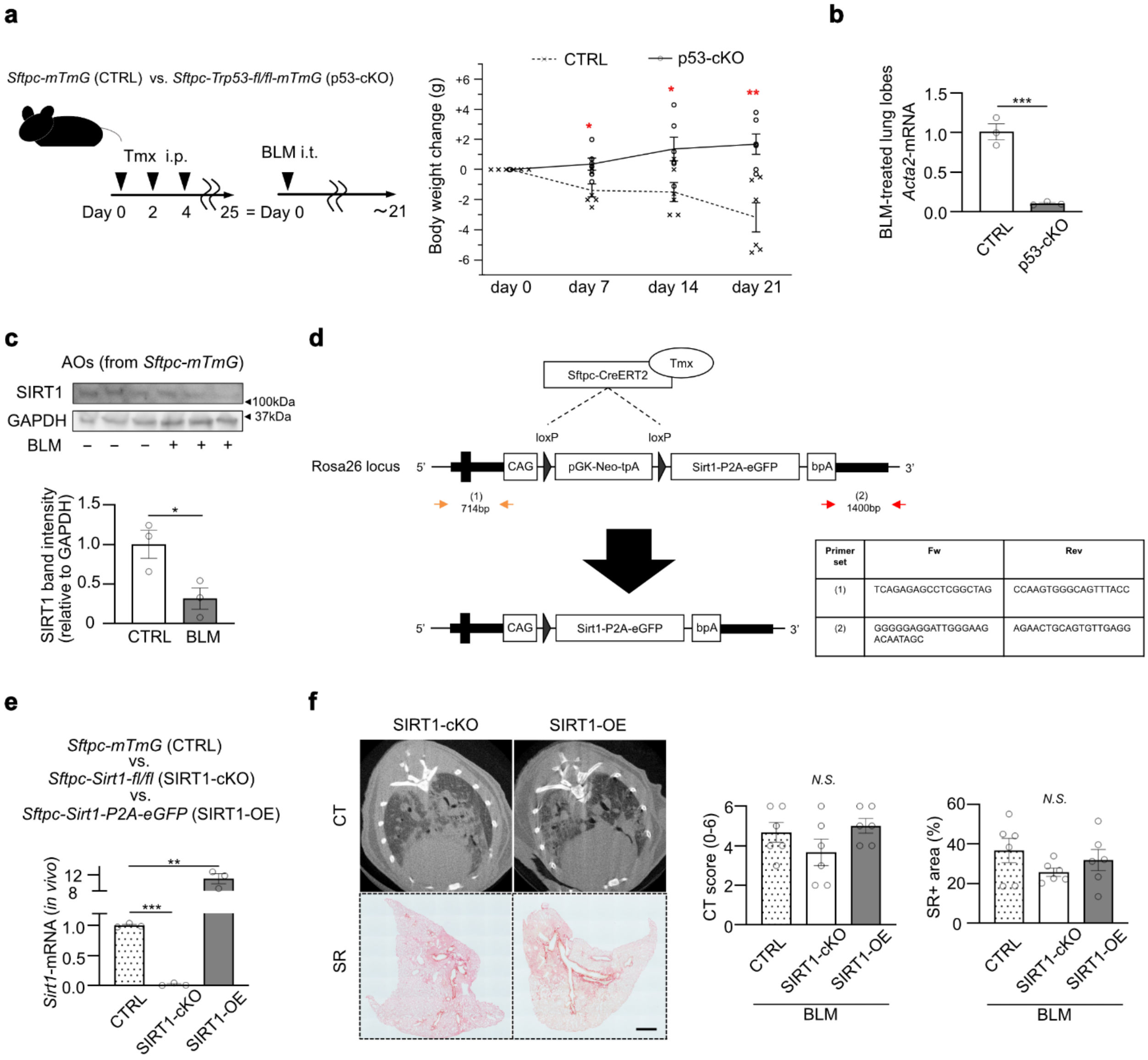

a) Diagram of BLM lung fibrosis model *in vivo* and changes in body weight that compare *Sftpc^{CreERT2}; Rosa26^{mTmG}* mice (CTRL) and *Sftpc^{CreERT2}; Trp53$^{flox/flox}$; Rosa26^{mTmG}* mice (p53-cKO). Data represent the mean ± SEM of results obtained from six independent mice in each group. **P* <0.05, ***P* <0.01 by comparing data in each timing (unpaired, two-tailed Student's t-test).

b) Comparison of mRNA expression level evaluated by qRT-PCR using *ex vivo* lung-lobe cells treated with BLM (100 μM, 24 h) on day 12. Data represent the mean ± SEM of the results obtained from three independent mice. ****P* <0.001 (unpaired, two-tailed Student's t-test).

c) Images of protein expression and quantification via western blotting for SIRT1 and GAPDH. The lanes were run on the same gel. The samples were derived from the same experiment and gels/blots were processed in parallel. The blots for GAPDH are same as Fig. 3c. Data represent the mean ± SEM of results obtained from three independent mice. **P* <0.05 (unpaired, two-tailed Student's t-test).

d) Targeting construct for making AT2-specific *Sirt1* conditional overexpression mice (*Sftpc^{CreERT2}; Rosa26$^{CAG-LSL-Sirt1-P2A-eGFP}$*).
e) Comparison of *Sirt1* mRNA expression level evaluated by qRT-PCR using primary AT2 cells isolated from each mouse line. Data represent the mean ± SEM of the results obtained from three independent mice. ***P* <0.01, ****P* <0.001 compared to CTRL (unpaired, two-tailed Student's t-test).
f) Representative images and the quantification of lung mCT and lung sections for SR staining (*scale bar: 1 mm*). Data represent the mean ± SEM of the results obtained from six independent mice. Statistical analyses are performed using unpaired, two-tailed Student's t-test (compared to CTRL).

Supplementary Fig. 5. Supporting data of Fig. 5.

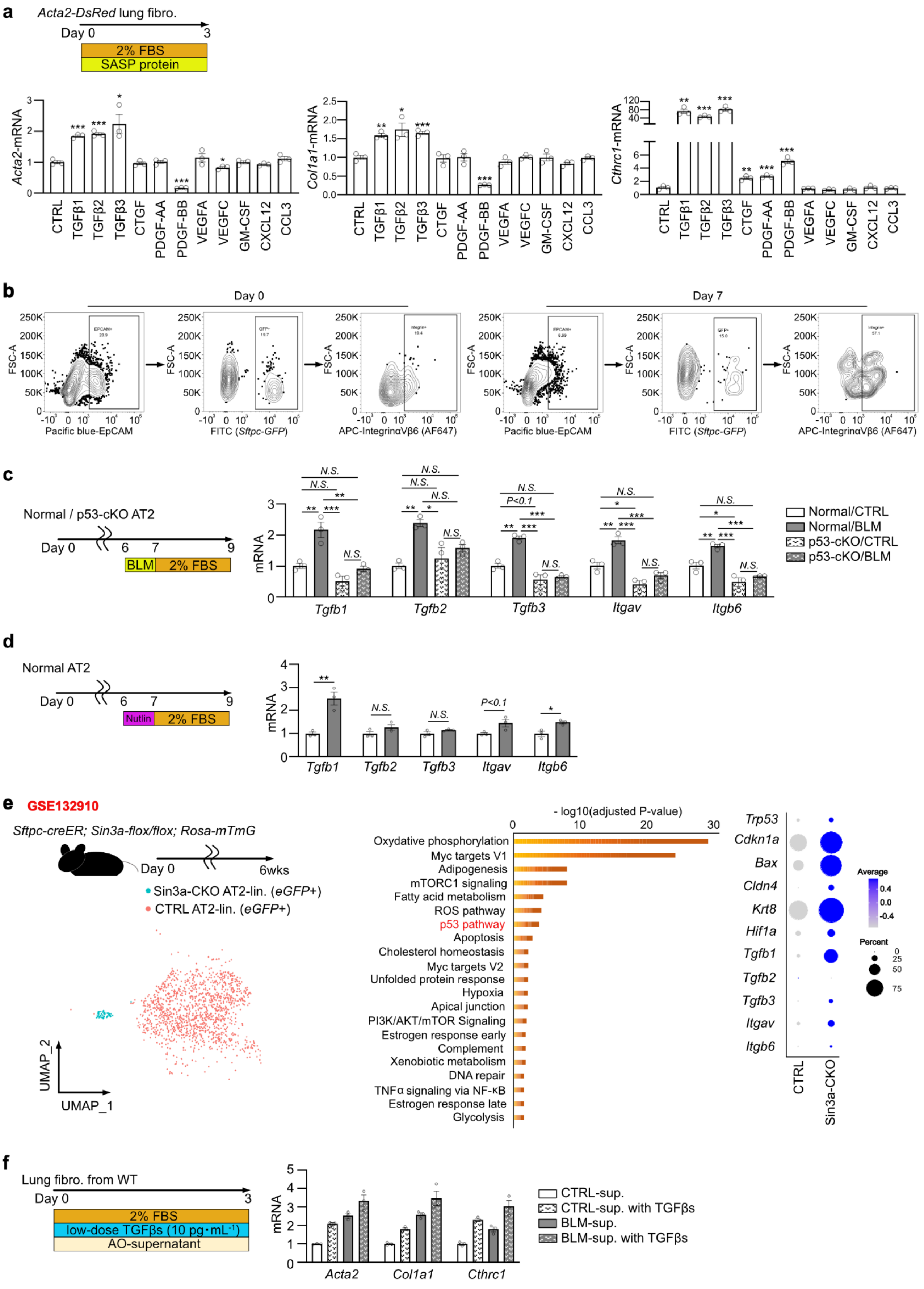

a) Diagram of experiment and comparison of mRNA expression levels evaluated by qRT-PCR using primary lung fibroblasts from *Acta2-DsRed* mice. Data represent the mean ± SEM of the results obtained from three independent mice. **P* <0.05, ***P* <0.01, ****P* <0.001 (compared to control; unpaired, two-tailed Student's t-test).
b) FACS gating strategy for integrin αVβ6 positivity using GFP^{+} AT2-lineage cells (days 0 and 7, *in vivo*).
c) Diagram of experiment and comparison of mRNA expression levels (for TGF-β-related genes) evaluated by qRT-PCR using normal AOs from $Sftpc^{CreERT2}$; $Rosa26^{mTmG}$ mice and p53-cKO AOs from $Sftpc^{CreERT2}$; $Trp53^{flox/flox}$; $Rosa26^{mTmG}$ mice. AOs were treated with BLM (100 μM, 24 h) and the sampling was done at culture day 9. Data represent the mean ± SEM of the results obtained from three independent mice. **P* <0.05, ***P* <0.01, ****P* <0.001 (one-way analysis of variance with Bonferroni correction).
d) Diagram of experiment and comparison of mRNA expression levels (for TGF-β-related genes) evaluated by qRT-PCR using normal AOs from $Sftpc^{CreERT2}$; $Rosa26^{mTmG}$ mice. AOs were treated with Nutlin-3a (2 μM, 24 h) and the sampling was done at culture day 9. Data represent the mean ± SEM of the results obtained from three independent mice. **P* <0.05, ***P* <0.01 (unpaired, two-tailed Student's t-test).
e) Reanalysis of single cell RNA-seq data of mouse lung AT2-lineage cells isolated from Sin3a-cKO mice (GSE132910). Clustering of AT2-lineage subpopulations (*left*), enriched pathways using upregulated genes in Sin3a-null AT2-lineage subpopulation (*middle*), and dot plot analysis for several target genes between the two subpopulations (*right*) are shown. *P* values are evaluated using the Fisher's exact test that assumes a binomial distribution and independence for probability of any gene belonging to any set.
f) Diagram of the experiments and comparison of mRNA expression levels evaluated by qRT-PCR using primary lung fibroblasts. Lung fibroblasts were isolated from wild-type mice and were treated with low-dose mixed TGF-β1/β2/β3 (10 $pg \cdot mL^{-1}$ each) and culture supernatants from normal AOs (from $Sftpc^{CreERT2}$; $Rosa26^{mTmG}$ mice) with or without BLM (100 μM, 24 h) pre-treatment. Data represent the mean ± SEM of results obtained from three independent mice.

Supplementary Fig. 6. Supporting data of Fig. 7 (1).

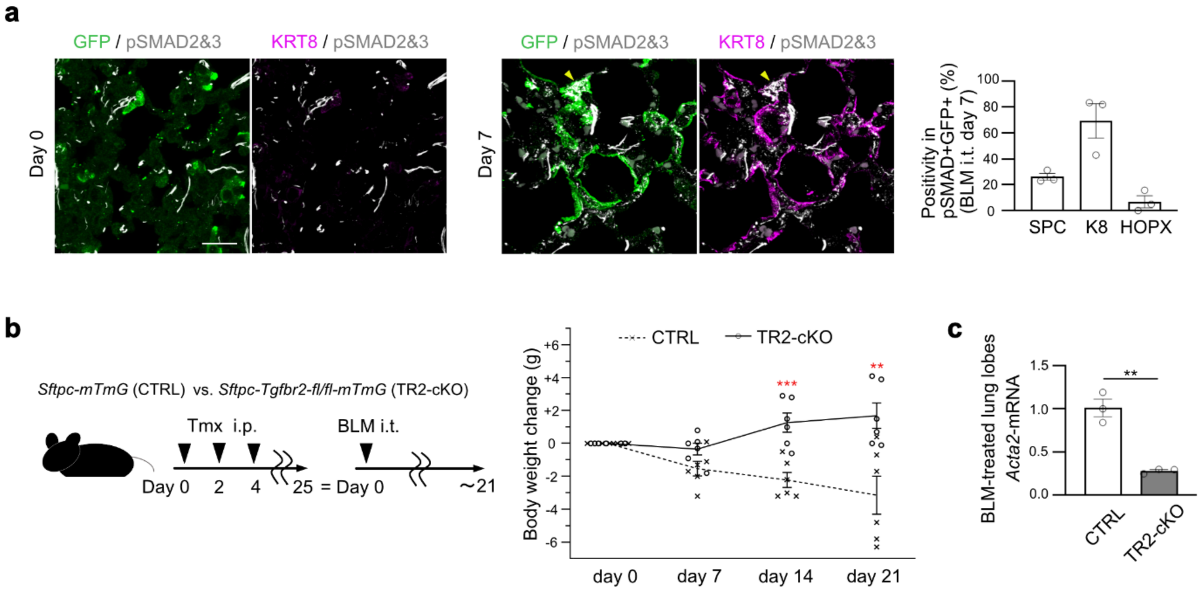

a) Representative images of immunostaining (*scale bar: 20 µm*) and quantification. Lung sections from BLM-treated $Sftpc^{CreERT2}$; $Rosa26^{mTmG}$ mice were stained with anti-GFP, anti-phosphorylated SMAD (p-SMAD) 2&3, and anti-KRT8 antibodies. A triangle indicates $KRT8^{+}$p-SMAD2&$3^{+}GFP^{+}$ AT2-lineage cell. Data represent the mean ± SEM of results obtained from three independent mice.

b) Diagram of BLM-induced lung fibrosis model *in vivo* and changes in body weight that compare $Sftpc^{CreERT2}$; $Rosa26^{mTmG}$ mice (CTRL) and $Sftpc^{CreERT2}$; $Tgfbr2^{flox/flox}$; $Rosa26^{mTmG}$ mice (TR2-cKO). Data represent the mean ± SEM of results obtained from six independent mice in each group. ***P* <0.01, ****P* <0.001 by comparing data in each timing (unpaired, two-tailed Student's t-test).

c) Comparison of mRNA expression level evaluated by qRT-PCR using *ex vivo* lung-lobe cells treated with BLM (100 µM, 24 h) on day 12. Data represent the mean ± SEM of the results obtained from three independent mice. ***P* <0.01 (unpaired, two-tailed Student's t-test).

Supplementary Fig. 7. Supporting data of Fig. 7 (2).

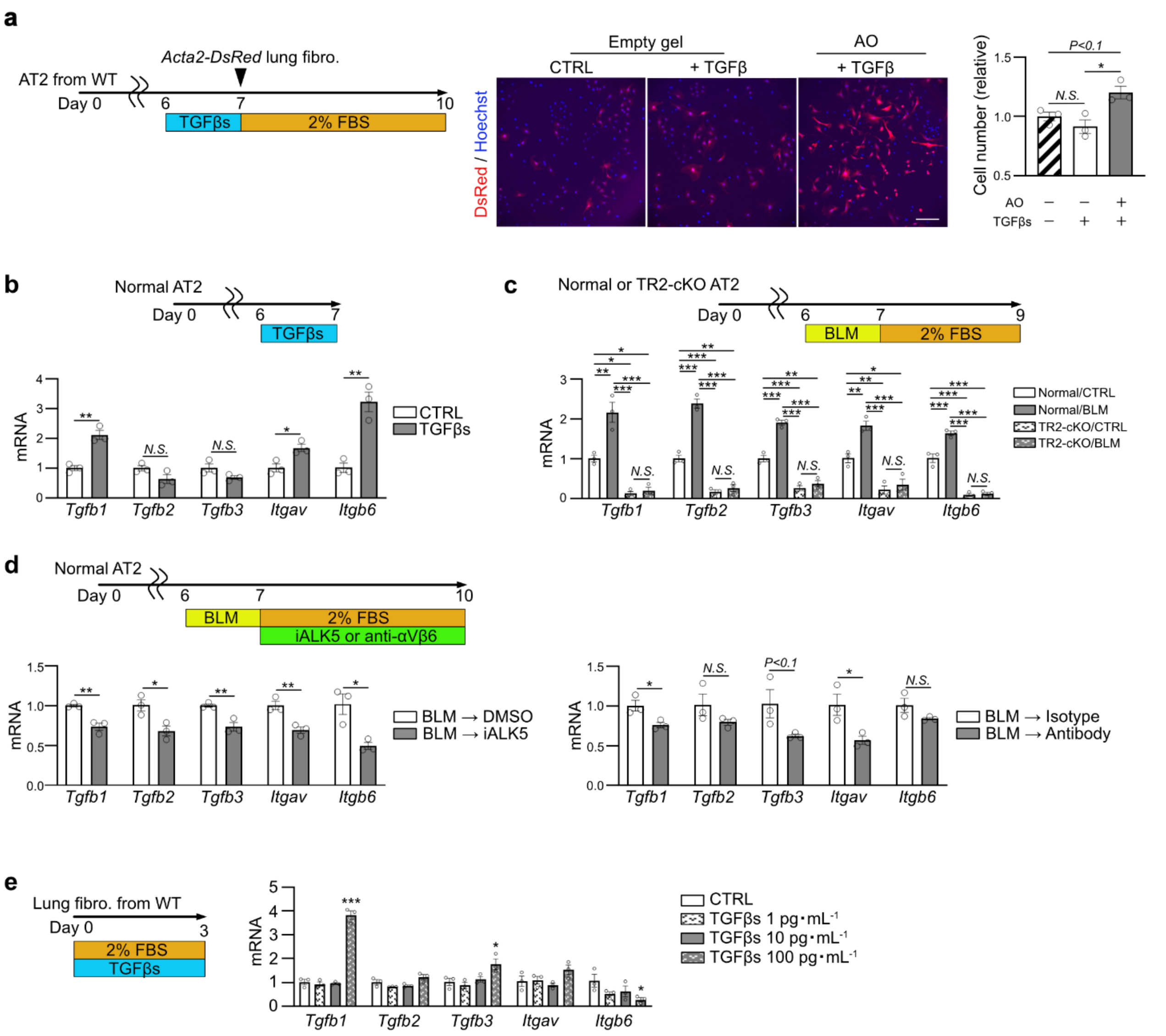

a) Representative images of co-cultured (myo)fibroblasts using normal AO treated with mixed TGF-β1/β2/β3 (5 pg·mL^{-1} each, 24 h) (*scale bar: 200 μm*) and quantification of the relative cell number around the empty or AO-containing gel. Data represent the mean ± SEM of results obtained from three independent mice. **P* <0.05 (one-way analysis of variance with Bonferroni correction).

b) Diagram of experiment and comparison of mRNA expression levels (for TGF-β-related genes) evaluated by qRT-PCR using normal AOs from *Sftpc^{CreERT2}; Rosa26^{mTmG}* mice. AOs were treated with mixed TGFβ1/β2/β3 (5 ng·mL^{-1} each, 24 h). Data represent the mean ± SEM of the results obtained from three independent mice. **P* <0.05, ***P* <0.01 (unpaired, two-tailed Student's t-test).

c) Diagram of experiment and comparison of mRNA expression levels (for TGF-β-related genes) evaluated by qRT-PCR using normal AOs from *Sftpc^{CreERT2}; Rosa26^{mTmG}* mice and TR2-cKO AOs from *Sftpc^{CreERT2}; Tgfbr2$^{flox/flox}$; Rosa26^{mTmG}* mice. AOs were treated with BLM (100 μM, 24 h) and the sampling was done at culture day 9. Data represent the mean ± SEM of the results obtained from three independent mice. **P* <0.05, ***P* <0.01, ****P* <0.001 (one-way analysis of variance with Bonferroni correction).

d) Diagram of experiment and the comparisons of mRNA expression levels (for TGF-β-related genes) evaluated by qRT-PCR using normal AOs (from *Sftpc^{CreERT2}; Rosa26^{mTmG}* mice) treated with an ALK5 inhibitor SB431542 (10 μM, 72 h) (*left*) or with a neutralizing antibody against integrin

$\alpha V\beta 6$ (100 μg·mL^{-1}, 72 h) (*right*), both after BLM administration (100 μM, 24 h). Data represent the mean ± SEM of the results obtained from three independent mice. **P* <0.05, ***P* <0.01 compared to each control situation (unpaired, two-tailed Student’s t-test).

e) Diagram of experiment and comparison of mRNA expression levels (for TGF-β-related genes) evaluated by qRT-PCR using primary lung fibroblasts from wild-type mice. Fibroblasts were treated with mixed TGF-β1/β2/β3 (1-100 pg·mL^{-1} each, 72 h). Data represent the mean ± SEM of the results obtained from three independent mice. **P* <0.05, ****P* <0.001 compared to CTRL (unpaired, two-tailed Student’s t-test).

Supplementary Fig. 8. Supporting data of Fig. 8.

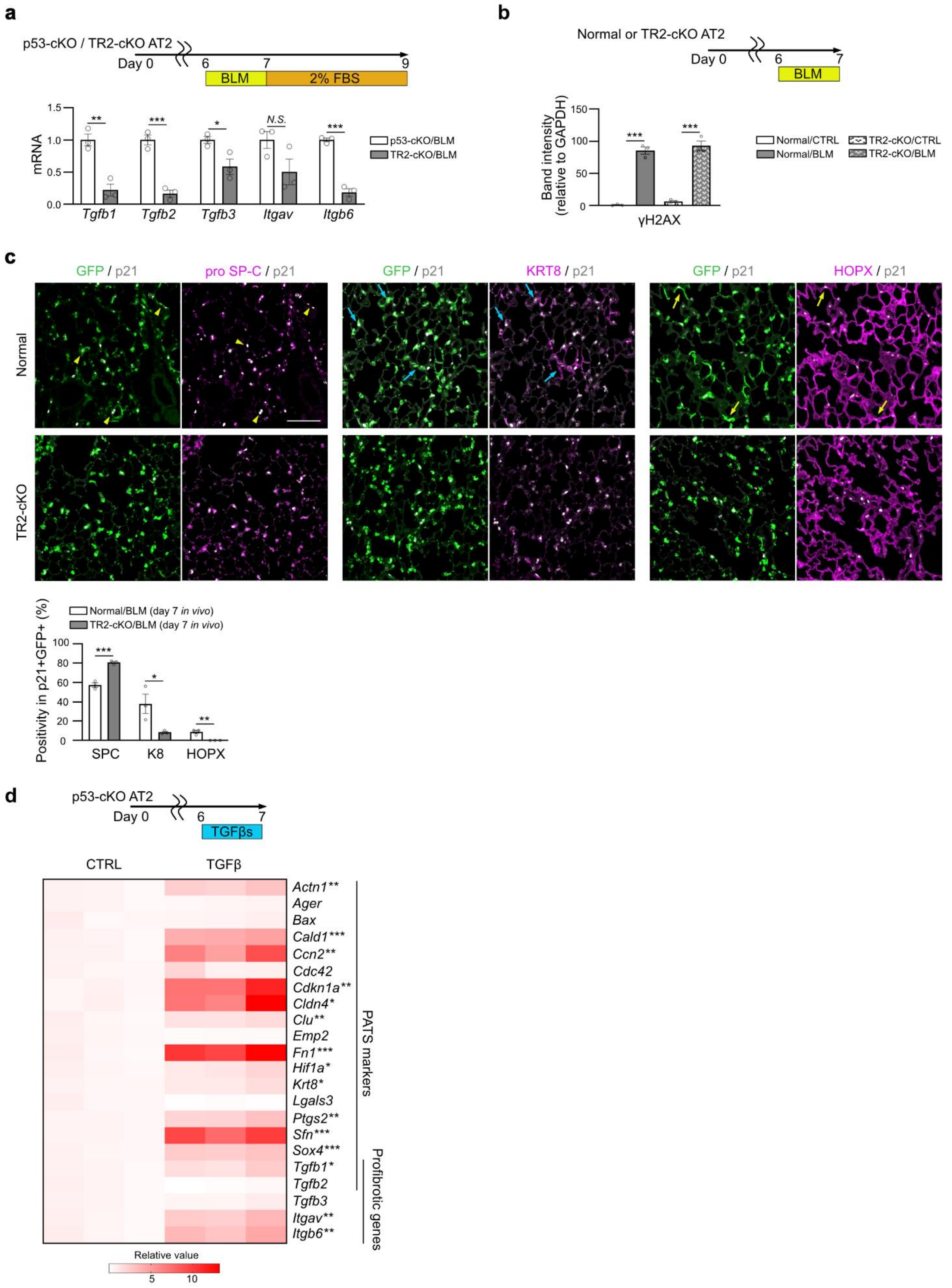

a) Diagram of experiment and comparison of mRNA expression levels (for TGF-β-related genes) evaluated by qRT-PCR using p53-cKO AOs from *Sftpc*^{CreERT2}*; Trp53*$^{flox/flox}$*; Rosa26*^{mTmG} mice and TR2-cKO AOs from *Sftpc*^{CreERT2}*; Tgfbr2*$^{flox/flox}$*; Rosa26*^{mTmG} mice. AOs were treated with BLM (100 μM, 24 h) and the sampling was done at culture day 9. Data represent the mean ± SEM of the results obtained from three independent mice. **P* <0.05, ***P* <0.01, ****P* <0.001 (one-way analysis of variance with Bonferroni correction). The original data is same as that in Fig. 8B.
b) Quantification of γH2AX protein expression of normal or TR2-cKO AOs treated with BLM (100 μM, 24 h) via western blotting. Data represent the mean ± SEM of results obtained from three independent mice analyzed using unpaired two-tailed Student's t-test (compared to each control).
c) Representative images of immunostaining (*scale bar: 100 μm*) and quantification. Lung sections from BLM-treated *Sftpc*^{CreERT2}*; Rosa26*^{mTmG} mice (CTRL) and *Sftpc*^{CreERT2}*; Tgfbr2*$^{flox/flox}$*; Rosa26*^{mTmG} mice (TR2-cKO) were stained with anti-GFP, anti-p21, and anti-pro SP-C/KRT8/HOPX antibodies, respectively. Yellow triangles, blue arrows, and yellow arrows indicate pro SP-C-negative, KRT8-positive, and HOPX-positive $p21^+GFP^+$ AT2-lineage cells in CTRL lungs, respectively. Data represent the mean ± SEM of results obtained from three independent mice (analyzed using unpaired two-tailed Student's t-test).
d) Diagram of experiment and heatmap visualization of mRNA expression levels (for PATS-related and profibrotic genes) evaluated by qRT-PCR using p53-cKO AOs. AOs were treated with mixed TGF-β1/β2/β3 (5 ng·mL^{-1} each, 24 h). Data were obtained from three independent mice. **P* <0.05, ***P* <0.01, ****P* <0.001 (unpaired, two-tailed Student's t-test).

Supplementary Fig. 9. Supporting data of Fig. 9.

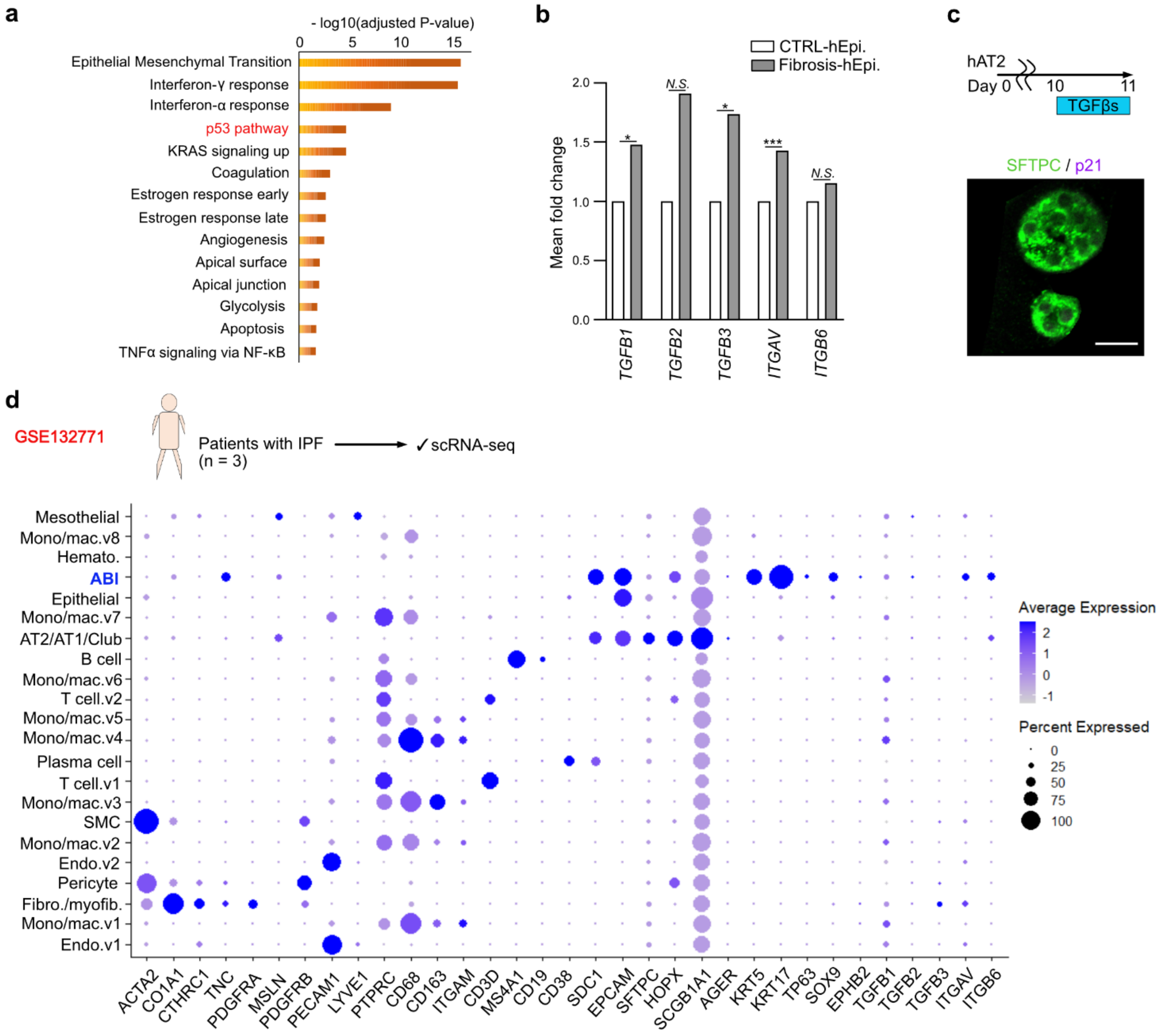

a) Enriched pathways of bulk RNA-seq data of human epithelial cells from pulmonary fibrosis patients using "MSigDB Hallmark 2020". Significantly upregulated 3160 genes (compared to epithelial cells from healthy subjects) are evaluated. *P* values are evaluated using the Fisher's exact test that assumes a binomial distribution and independence for probability of any gene belonging to any set.

b) Comparisons of mean fold change in each target gene. **P* <0.05, ****P* <0.001 (the exact adjusted *P* values are shown in a previous paper [Am J Respir Crit Care Med 199, 1517-1536, (2019)]).

c) Diagram of experiment and the representative image of human AO treated with mixed TGF-β1/β2/β3 (5 ng·mL^{-1} each, 24 h). The AO section was stained with anti-SFTPC antibody and anti-p21 antibody (*scale bar: 20 µm*).

d) Reanalysis of single cell RNA-seq data of lung samples isolated from patients with idiopathic pulmonary fibrosis (IPF). Dot plot analysis for several target genes between subpopulations is shown.

Supplementary Fig. 10. Supporting data of Discussion.

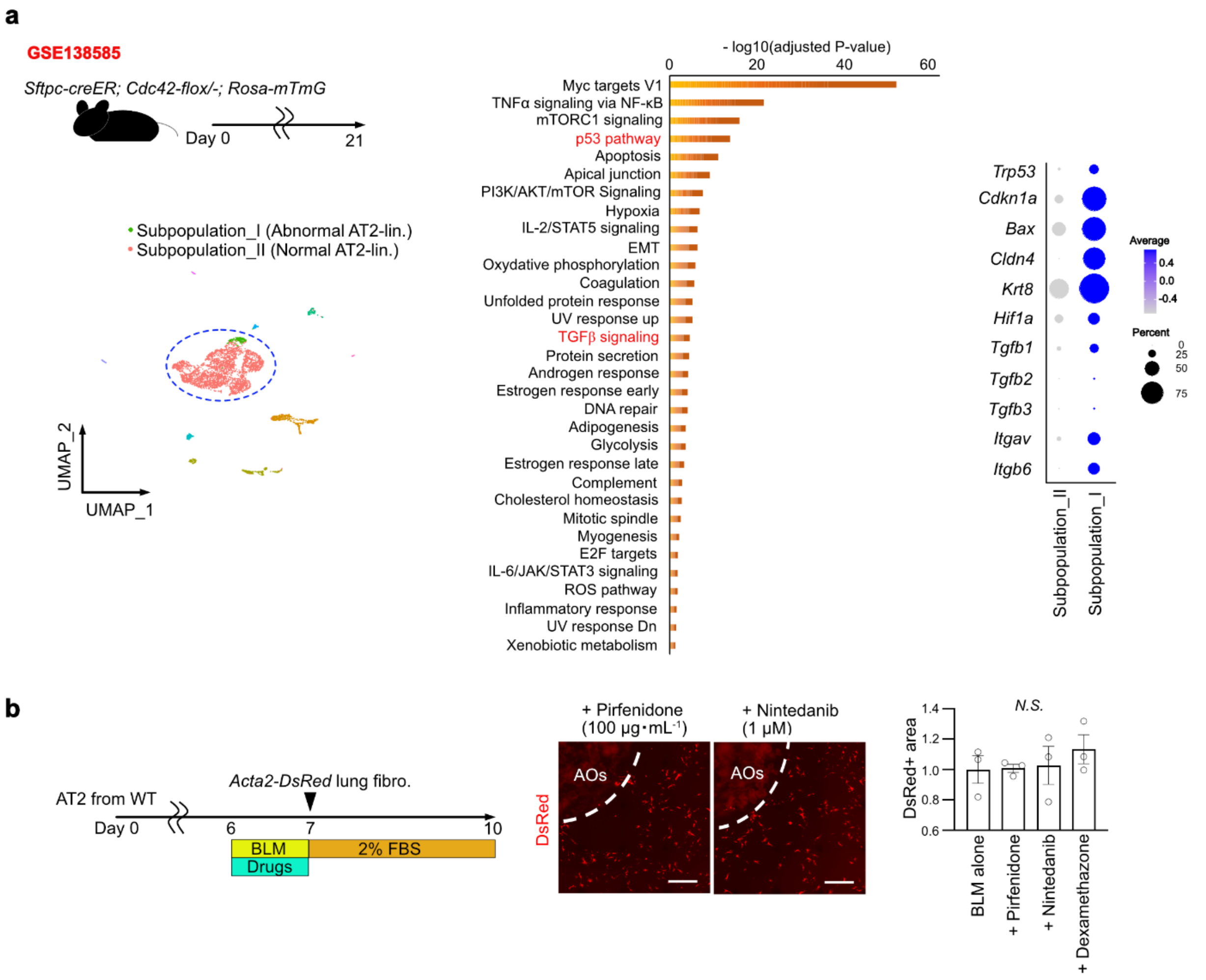

a) Reanalysis of single cell RNA-seq data of mouse lung AT2-lineage cells isolated from Cdc42-cKO mice (GSE138585). Clustering of AT2-lineage subpopulations (*left*), enriched pathways using upregulated genes in abnormal AT2-lineage subpopulation (*middle*), and dot plot analysis for several target genes between the two subpopulations (*right*) are shown. *P* values are evaluated using the Fisher's exact test that assumes a binomial distribution and independence for probability of any gene belonging to any set.

b) Diagram of experiment, representative images of co-cultured (myo)fibroblasts, and the quantification of relative DsRed^{+} area around the AO-containing gel (*scale bar: 1 mm*). AOs (from wild type mice) were treated with BLM (100 µM, 24 h) and drugs: pirfenidone (100 µg·mL^{-1}, 24 h), nintedanib (1 µM, 24h), or dexamethasone (50 ng·mL^{-1}, 24h). Data represent the mean ± SEM of the results obtained from three independent mice (analyzed using unpaired two-tailed Student's t-test and compared to BLM alone).
