# Supplementary material for: Autocrine TGF-β-positive feedback in profibrotic AT2-lineage cells plays a crucial role in non-inflammatory lung fibrogenesis
Source: Nat Commun. 2023 Aug 31;14:4956. doi: 10.1038/s41467-023-40617-y (PMC10471635; doi:10.1038/s41467-023-40617-y)
Supplement: Supplementary file 3 — Description of Additional Supplementary Files [file 41467_2023_40617_MOESM3_ESM.pdf]

Description of Additional Supplementary Files

Supplementary Data Legends

Supplementary Data 1: Major materials used in this study

Supplementary Data 2: Upregulated genes in bleomycin-treated alveolar organoids evaluated by RNA-seq.

Supplementary Data 3: Primer sequences used in this study

Supplementary Data 4: Peak-matched genes in ChIP-seq and commonly upregulated genes even in RNA-seq for bleomycin-treated alveolar organoids (BLM-AO)

Supplementary Movie Legends

Supplementary Movie 1: Live imaging for co-culture of control alveolar organoids (AOs) with primary fibroblasts isolated from Acta2-DsRed mice. Imaging was performed every 1.5 h until 72 h. AOs in Matrigel are located in the left upper area.

Supplementary Movie 2: Live imaging for co-culture of bleomycin-treated AO with primary fibroblasts isolated from Acta2-DsRed mice. Imaging was performed every 1.5 h until 72 h. AOs in Matrigel are located in the left upper area.
