# Supplementary material for: Autocrine TGF-β-positive feedback in profibrotic AT2-lineage cells plays a crucial role in non-inflammatory lung fibrogenesis
Source: Nat Commun. 2023 Aug 31;14:4956. doi: 10.1038/s41467-023-40617-y (PMC10471635; doi:10.1038/s41467-023-40617-y)
Supplement: Supplementary file 10 — Reporting Summary [file 41467_2023_40617_MOESM10_ESM.pdf]

nature portfolio

Corresponding author(s): Mitsuru Morimoto

Last updated by author(s): 2023-07-10

Reporting Summary

Nature Portfolio wishes to improve the reproducibility of the work that we publish. This form provides structure for consistency and transparency in reporting. For further information on Nature Portfolio policies, see our Editorial Policies and the Editorial Policy Checklist.

Please do not complete any field with "not applicable" or n/a. Refer to the help text for what text to use if an item is not relevant to your study.
For final submission: please carefully check your responses for accuracy; you will not be able to make changes later.

Statistics

For all statistical analyses, confirm that the following items are present in the figure legend, table legend, main text, or Methods section.

n/a	Confirmed	
☐	☑	The exact sample size (*n*) for each experimental group/condition, given as a discrete number and unit of measurement
☐	☑	A statement on whether measurements were taken from distinct samples or whether the same sample was measured repeatedly
☐	☑	The statistical test(s) used AND whether they are one- or two-sided<br>*Only common tests should be described solely by name; describe more complex techniques in the Methods section.*
☑	☐	A description of all covariates tested
☐	☑	A description of any assumptions or corrections, such as tests of normality and adjustment for multiple comparisons
☐	☑	A full description of the statistical parameters including central tendency (e.g. means) or other basic estimates (e.g. regression coefficient) AND variation (e.g. standard deviation) or associated estimates of uncertainty (e.g. confidence intervals)
☑	☐	For null hypothesis testing, the test statistic (e.g. *F*, *t*, *r*) with confidence intervals, effect sizes, degrees of freedom and *P* value noted<br>*Give P values as exact values whenever suitable.*
☑	☐	For Bayesian analysis, information on the choice of priors and Markov chain Monte Carlo settings
☑	☐	For hierarchical and complex designs, identification of the appropriate level for tests and full reporting of outcomes
☑	☐	Estimates of effect sizes (e.g. Cohen's *d*, Pearson's *r*), indicating how they were calculated

Our web collection on statistics for biologists contains articles on many of the points above.

Software and code

Policy information about availability of computer code

Data collection	Images were taken by Zeiss 710 confocal microscope (Carl Zeiss) or IX83 microscope (OLYMPUS). qPCR analyses were performed using QuantStudio 5. FACSDiva v8.0.2 (BD Bioscineces) was used to collect FACS data.
Data analysis	Images were analyzed by Fiji v1.53 Software. Statistical analyses were done using Excel 2019 (Microsoft) or EZR v1.52 (under R v4.0.2) software.<br>For RNA-seq, gene expression quantification was performed using StringTie v2.1.4, and normalization was performed using edgeR v3.32.1. Likelihood ratio tests for differential expression were performed with the edgeR glmFit and glmLRT functions, and FDR was calculated using the Benjamini-Hochberg method.<br>For ChIP-seq, peaks were called using MACS3 ver. 3.0.0b16 with the significance cut-off q-value = 0.01 for each sample. Identification of peaks that were significantly enriched in the BLM-treated samples was calculated using the DiffBind v3.8.47 with edgeR v3.40.28 and filtered with FDR <0.05. To associate peaks with genes, peak positions were converted to mm10 and then analyzed on the GREAT v4.0.4 website. FACS data were analyzed using FlowJo v10.8.1.

For manuscripts utilizing custom algorithms or software that are central to the research but not yet described in published literature, software must be made available to editors and reviewers. We strongly encourage code deposition in a community repository (e.g. GitHub). See the Nature Portfolio guidelines for submitting code & software for further information.

Data

Policy information about availability of data

All manuscripts must include a data availability statement. This statement should provide the following information, where applicable:

- Accession codes, unique identifiers, or web links for publicly available datasets
- A description of any restrictions on data availability
- For clinical datasets or third party data, please ensure that the statement adheres to our policy

The authors declare that all data supporting the findings of this study are available within the article and its supplementary materials, including a Source Data file. The raw images are available in the System Science of Biological Dynamics (SSBD) repository (URL: https://ssbd.riken.jp/repository/288/; DOI: https://doi.org/10.24631/ssbd.repos.2023.04.288). Our original sequencing data in this study are available at NCBI GEO under the accession numbers GSE211531 [https://www.ncbi.nlm.nih.gov/geo/query/acc.cgi?acc=GSE211531] (RNA-seq) and GSE231445 [https://www.ncbi.nlm.nih.gov/geo/query/acc.cgi?acc=GSE231445] (ChIP-seq). For RNA-seq, the trimmed RNA-seq reads were aligned to the UCSC GRCm39/mm39 genome sequences. For ChIP-seq, the trimmed reads were aligned to the UCSC mm39 genome sequences.
Previously published sequencing data that were reanalyzed are available under the accession number GSE138585 [https://www.ncbi.nlm.nih.gov/geo/query/acc.cgi?acc=GSE138585]. GSE132910 [https://www.ncbi.nlm.nih.gov/geo/query/acc.cgi?acc=GSE132910], and GSE132771 [https://www.ncbi.nlm.nih.gov/geo/query/acc.cgi?acc=GSE132771].

Research involving human participants, their data, or biological material

Policy information about studies with human participants or human data. See also policy information about sex, gender (identity/presentation), and sexual orientation and race, ethnicity and racism.

Reporting on sex and gender	Sex and/or gender was not considered.
Reporting on race, ethnicity, or other socially relevant groupings	Only Japanese patients are included.
Population characteristics	Three persons: 49 y.o. female; 78 y.o. male; 48 y.o. male.
Recruitment	Patients at Kobe University Hospital who received surgery for suspected early-stage lung cancer and did not have apparent emphysematous nor fibrotic changes on their CT images were recruited.
Ethics oversight	The collection and use of human lung samples has been approved by the Institutional Review Board of Kobe University Graduate School of Medicine (B2056707). Informed consent has been obtained from all the patients.

Note that full information on the approval of the study protocol must also be provided in the manuscript.

Field-specific reporting

Please select the one below that is the best fit for your research. If you are not sure, read the appropriate sections before making your selection.

☑ Life sciences ☐ Behavioural & social sciences ☐ Ecological, evolutionary & environmental sciences

For a reference copy of the document with all sections, see nature.com/documents/nr-reporting-summary-flat.pdf

Life sciences study design

All studies must disclose on these points even when the disclosure is negative.

Sample size	Sample size was decided based on the standard in the field.
Data exclusions	No data were excluded in this study.
Replication	The number of replicates was described in the legends of Figures.
Randomization	Randomization was not applicable in this study and samples were chosen based on genotype.
Blinding	Formal blinding was not done. We treated and analyzed all samples in the similar procedure.

Reporting for specific materials, systems and methods

We require information from authors about some types of materials, experimental systems and methods used in many studies. Here, indicate whether each material, system or method listed is relevant to your study. If you are not sure if a list item applies to your research, read the appropriate section before selecting a response.

Materials & experimental systems

n/a	Involved in the study	
☐	☑	Antibodies
☑	☐	Eukaryotic cell lines
☑	☐	Palaeontology and archaeology
☐	☑	Animals and other organisms
☑	☐	Clinical data
☑	☐	Dual use research of concern
☑	☐	Plants

Methods

n/a	Involved in the study	
☐	☑	ChIP-seq
☐	☑	Flow cytometry
☑	☐	MRI-based neuroimaging

Antibodies

Antibodies used

**Antibodies for immunostaining**	**Supplyer**	**Catalog number**
Anti-SFTPC antibody: rabbit, polyclonal	GeneTex	#GTX54694
Anti-ProSP-C antibody: rabbit, polyclonal	Millipore	#AB3786
Anti-αSMA antibody: mouse, monoclonal <clone 1A4>	Dako	#M0851
Anti-Gamma H2AX (p Ser139) antibody: rabbit, polyclonal	Novus Biologicals	#NB100-384
Anti-GFP antibody: chicken, polyclonal	Thermo Fisher Scientific	#A10262
Anti-p53 antibody: mouse, monoclonal <clone 1C12>	Cell Signaling	#2524
Anti-p21 antibody (for mouse samples): rabbit monoclonal <clone EPR18021>	Abcam	#ab188224
Anti-p21 antibody (for human samples): goat polyclonal	R&D	#AF1047
Anti-Phospho-Smad2(Ser465/467)/Smad3(Ser423/425) antibody: rabbit monoclonal <clone D27F4>	Cell Signaling	#8828
Anti-RAGE antibody: goat polyclonal	R&D	#AF1145
Anit-Cytokeratin 8 antibody: rat monoclonal <clone TROMA-1>	DSHB	#TROMA-I
Anti-HOP (FL-73) antibody: rabbit polyclonal	SANTA CRUZ	#sc-30216
Anti-Ki-67 antibody: mouse monoclonal <clone B56>	BD	#550609
Anti-ERG (EPR3864) antibody: rabbit monoclonal <clone EPR3864>	Abcam	#ab92513
**Antibodies for FACS/MACS**		
Anti-CD326 (EpCAM) antibody, APC: rat monoclonal <clone G8.8>	Invitrogen	#17-5791-80
Anti-CD326 (EpCAM) antibody, eFluor® 450: rat monoclonal <clone G8.8>	Invitrogen	#48-5791-82
Anti-MHC Class II (I-A/I-E) antibody, eFluor® 450: rat monoclonal <clone M5/114.15.2>	eBioscience	#48-5321
Anti-CD31 antibody, PE/Cy7: rat monoclinal <clone 390>	BioLegend	#102417
Anti-CD31 antibody, APC: rat monoclinal <clone 390>	BioLegend	#102409
Anti-CD45 antibody, PE-Cy7: rat monoclonal <clone 30-F11>	eBioscience	#25-0451
Anti-CD45 antibody, APC: rat monoclonal <clone 30-F11>	BioLegend	#103111
Anti-CD140a antibody, PE: rat monoclonal <clone APA5>	BioLegend	#135905
Anti-LYVE-1 antibody, APC: rat monoclonal <clone 223322>	R&D Systems	#FAB2125A
Anti-CD146 antibody: APC: rat monoclonal <clone ME-9F1>	BioLegend	#134711
Anti-HT2-280 antibody: mouse monoclonal	TERRACE BIOTECH	#TB-27AHT2-280
**Antibodies for western blotting**		
Anti-Gamma H2AX (p Ser139) antibody: rabbit polyclonal	Novus Biologicals	#NB100-384
Anti-p53 antibody: mouse monoclonal <clone 1C12>	Cell Signaling	#2524T
Anti-Acetylated-p53 (Lys379) antibody: rabbit polyclonal	Cell Signaling	#2570S
Anti-p21 antibody: rabbit monoclonal <clone EPR18021>	Abcam	#ab188224
Anti-Smad2 antibody: rabbit monoclonal <clone ARC0343>	ABclonal	#A19114
Anti-Phospho-Smad2 (Ser465/467) antibody: rabbit monoclonal <clone 138D4>	Cell Signaling	#3108T
Anti-Smad3 antibody: rabbit monoclonal <clone ARC53861>	ABclonal	#A19115
Anti-Phospho-Smad3 (Ser423/425) antibody: rabbit monoclonal <clone C25A9>	Cell Signaling	#9520T
Anti-p16INK4A antibody: rabbit monoclonal <clone E5F3Y>	Cell Signaling	#29271
Anti-p19ARF antibody: rabbit monoclonal <clone E9X4Z>	Cell Signaling	#77184
Anti-MDM2 antibody: rabbit monoclonal <clone E3G5I>	Cell Signaling	#51541
Anti-CKAP4 (p63) antibody, rabbit polyclonal	Invitrogen	#PA5-96803
Anti-SIRT1 antibody: mouse monoclonal <clone 19A7AB4>	Abcam	#ab110304
Anti-GAPDH antibody: mouse monoclonal <clone 6C5>	Abcam	#ab8245
Anti-rabbit IgG, HRP-linked antibody	Cell Signaling	#7074P2
Peroxidase-AffiniPure Donkey Anti-Mouse IgG (H+L)	Jackson ImmunoResearch Laboratories	#715-035-151
**Antibodies for ChIP-seq**		
Anti-p53 antibody: rabbit monoclonal <clone D2H9O>	Cell Signaling	#32532
Dynabeads M-280 Sheep Anti-Mouse IgG	Thermo Fisher Scientific	#11201D

Validation

All antibodies are commercially available and have been validated by the manufacturers.

Animals and other research organisms

Policy information about studies involving animals; ARRIVE guidelines recommended for reporting animal research, and Sex and Gender in Research

Laboratory animals	All mice (male, 8–12 week-old) were bred and housed in a specific pathogen-free mouse facility at constant temperature (18–23 °C) and humidity (40–60%) in sterilized plastic cages. A 12h-light/12h-dark cycle was used. Sftpc-CreERT2 (B6.129S-Sftpctm1(cre/ERT2)Blh/J) (#028054), Rosa26-mTmG (B6.129(Cg)-Gt(ROSA)26Sortm4(ACTB-tdTomato,-EGFP)Luo/J) (#007676), Tgfbr2-flox (B6;129-Tgfbr2tm1Karl/J) (#012603), Acta2-DsRed (C.FVB-Tg(Acta2-DsRed)1Rkl/J) (#031159), and Sirt1-flox (B6.129-Sirt1tm3Fwa/DsinJ) (#029603) mice were purchased from The Jackson Laboratory. Pdgfra-creERT2 mice were kindly gifted by Dr. Brigid Hogan and Dr. Christina E. Barkauskas. Trp53-flox (C57BL/6N-Trp53<em1Rbrc>) (#RBRC09921) mice were provided from RIKEN-BRC. Sirt1 conditional overexpression mice (Rosa26CAG-LSL-Sirt1-P2A-eGFP) (Accession No. CDB0120E: https://large.riken.jp/distribution/mutant-list.html) were newly established with CRISPR/Cas9-mediated genome editing in C57BL/6 mice zygotes at RIKEN-BDR. All mice were maintained on a C57BL/6 background.
Wild animals	No wild animals were used in this study.
Reporting on sex	Only male mice were used.
Field-collected samples	No field-collected samples were used.
Ethics oversight	All mouse experiments were approved by the Institutional Animal Care and Use Committee of RIKEN Kobe Branch. Mice were handled in accordance with the ethics guidelines of the institute.

Note that full information on the approval of the study protocol must also be provided in the manuscript.

ChIP-seq

Data deposition

- [x] Confirm that both raw and final processed data have been deposited in a public database such as GEO.
- [x] Confirm that you have deposited or provided access to graph files (e.g. BED files) for the called peaks.

Data access links<br>*May remain private before publication.*	GSE231445 (https://www.ncbi.nlm.nih.gov/geo/query/acc.cgi?acc=GSE231445)
Files in database submission	P621_15_1_S9_R1_001.fastq.gz; P621_07_1_S1_R1_001.fastq.gz; P621_19_1_S13_R1_001.fastq.gz; P621_11_1_S5_R1_001.fastq.gz;<br>P621_16_1_S10_R1_001.fastq.gz;<br>P621_08_1_S2_R1_001.fastq.gz; P621_20_1_S14_R1_001.fastq.gz; P621_12_1_S6_R1_001.fastq.gz; P621_15_1_R1.filtered.bam;<br>P621_07_1_R1.filtered.bam<br>P621_19_1_R1.filtered.bam; P621_11_1_R1.filtered.bam; P621_16_1_R1.filtered.bam; P621_08_1_R1.filtered.bam; P621_20_1_R1.filtered.bam;<br>P621_12_1_R1.filtered.bam<br>1-Cd1v1_peaks.narrowPeak; 5-Cd1v2_peaks.narrowPeak; 2-Bd1v1_peaks.narrowPeak; 6-Bd1v2_peaks.narrowPeak; BLM_enriched_peaks.bed;<br>BLM_enriched_genes_mm10.tx
Genome browser session<br>(e.g. UCSC)	n/a

Methodology

Replicates	All ChIP-seq samples were completed in duplicate.
Sequencing depth	Sequencing was single-end, 101bp reads, at a sequencing depth of approximately 34 million, >74% of reads mapped uniquely on average.
Antibodies	Anti-p53 rabbit monoclonal antibody (Cell Signaling; #32532), Lot: 2, 177 µg/mL.
Peak calling parameters	The trimmed reads were aligned to the UCSC mm39 genome sequences using Bowtie2 ver. 2.5.1 with default parameters. Filtering of uniquely mapped reads was performed using -F '[XS] == null and not unmapped and not duplicate' in Sambamba ver. 0.8.2. Peaks were called using MACS3 ver. 3.0.0b1 with the significance cut-off q-value = 0.01 for each sample. Identification of peaks that were significantly enriched in the BLM-treated samples was calculated using the DiffBind ver. 3.8.4 with edgeR ver. 3.40.2 and filtered with FDR < 0.05.
Data quality	Sample name, Peak number FDR 5% and 5-fold<br>Alveolar_organoids_CTRL_rep1, 42257; Alveolar_organoids_CTRL_rep2, 41850; Alveolar_organoids_BLM_rep1, 32675; Alveolar_organoids_BLM_rep2, 34167
Software	Adapter and low-quality trimming: Trim galore ver. 0.6.7, Genome mapping: Bowtie2 ver. 2.5.1 and mm39 genome sequences,<br>Filtering of uniquely-mapped reads: Sambamba ver. 0.8.2, Peak calling: MACS3 ver. 3.0.0b1, Differential enriched peaks: DiffBind ver. 3.8.4 with edgeR<br>ver. 3.40.2, Enrich analysis: GREAT ver. 4.0.4 and Enrichr with MSigDB database.

Flow Cytometry

Plots

Confirm that:

☑ The axis labels state the marker and fluorochrome used (e.g. CD4-FITC).

☑ The axis scales are clearly visible. Include numbers along axes only for bottom left plot of group (a 'group' is an analysis of identical markers).

☑ All plots are contour plots with outliers or pseudocolor plots.

☑ A numerical value for number of cells or percentage (with statistics) is provided.

Methodology

Sample preparation	Mice were sacrificed by carbon dioxide and perfused with 5 mL of saline solution thorough the right ventricle. 0.8 mL of protease solution (Collagenase type I (450 U/mL, Worthington), Elastase (1 U/mL, Worthington), Dispase (5 U/mL, Corning), DNaseI (0.05 mg/mL, SIGMA), Trypsin (0.025%, Thermo Fisher Scientific) in DMEM/F12) was intratracheally injected and the trachea was tied with a string.<br>After incubation for 5 min at 37 °C on a dish, only lung lobes were extracted. These lobes were chopped to small pieces with a sterile blade and incubated in a total of 4 mL of the protease solution for 30 min at 37 °C with a rotator.<br>Then the tissues/cells were dissociated by repetitive pipetting and 4 mL of PBS containing 10 % FBS was added. And then cells were filtered through 100 μm and 40 μm strainer. After a centrifugation (400g, 5 min), the cells were incubated with RBC lysis buffer (BioLegend) at room temperature for 3 min.<br>Next, the cells were resuspended in PBS containing 3 % FBS and centrifuged (400g, 5 min). After that, the cells were stained with antibodies and sorted.
Instrument	Analysis and cell sorting were carried out on a FACS AriaII machine (BD Bioscience).
Software	FACSDiva v8.0.2 (BD Bioscineces) was used to collect data and FlowJo v10.8.1 was used to analyze all FACS data.
Cell population abundance	The purity of post-sort cells was not evaluated.
Gating strategy	Primary hematopoietic cells from mouse lungs: morphology (FSC/SSC) -> single cells (FSC-W/FSC-H) -> CD45-PE-Cy7+<br>Primary alveolar epithelial type 2 cells from Sftpc-creERT2; Rosa-mTmG mice: morphology (FSC/SSC) -> single cells (FSC-W/FSC-H) -> GFP+ -> 7-AAD- (live)<br>Primary alveolar epithelial type 2 cells from wild-type mice: morphology (FSC/SSC) -> single cells (FSC-W/FSC-H) -> 7-AAD- (live) -> CD45&CD31-PE-Cy7- -> EpCAM-APC<br>+ & Pacific blue- MHC class II<br>Primary lung fibroblasts: morphology (FSC/SSC) -> single cells (FSC-W/FSC-H) -> DAPI- (live) -> APC-EpCAM&CD45&CD31&CD146&LYVE1+ & CD140a-PE+<br>Boundaries between “positive” and “negative” populations were defined using fluorescence minus one sample, isotype control, and/or wild-type sample.

☑ Tick this box to confirm that a figure exemplifying the gating strategy is provided in the Supplementary Information.

This checklist template is licensed under a Creative Commons Attribution 4.0 International License, which permits use, sharing, adaptation, distribution and reproduction in any medium or format, as long as you give appropriate credit to the original author(s) and the source, provide a link to the Creative Commons license, and indicate if changes were made. The images or other third party material in this article are included in the article's Creative Commons license, unless indicated otherwise in a credit line to the material. If material is not included in the article's Creative Commons license and your intended use is not permitted by statutory regulation or exceeds the permitted use, you will need to obtain permission directly from the copyright holder. To view a copy of this license, visit http://creativecommons.org/licenses/by/4.0/
